# Complete chloroplast genomes of *Sorbus *sensu stricto (Rosaceae): comparative analyses and phylogenetic relationships

**DOI:** 10.1186/s12870-022-03858-5

**Published:** 2022-10-22

**Authors:** Chenqian Tang, Xin Chen, Yunfei Deng, Liyang Geng, Jianhui Ma, Xueyan Wei

**Affiliations:** 1 Co-Innovation Center for Sustainable Forestry in Southern China, College of Biology and the Environment, Nanjing Forestry University, Nanjing, 210037 Jiangsu China; 2 Key Laboratory of Plant Resources Conservation and Sustainable Utilization, South China Botanical Garden, Chinese Academy of Sciences, Guangzhou, 510650 China

**Keywords:** *Sorbus* sensu stricto, Chloroplast genome, Repeats, Codon usage, Sequence divergence, Phylogeny

## Abstract

**Background:**

*Sorbus *sensu stricto (*Sorbus s.s.*) is a genus with important economical values because of its beautiful leaves, and flowers and especially the colorful fruits. It belongs to the tribe Maleae of the family Rosaceae, and comprises about 90 species mainly distributed in China. There is on-going dispute about its infrageneric classification and species delimitation as the species are morphologically similar. With the aim of shedding light on the circumscription of taxa within the genus, phylogenetic analyses were performed using 29 *Sorbus s.s.* chloroplast (cp) genomes (16 newly sequenced) representing two subgenera and eight sections.

**Results:**

The 16 cp genomes newly sequenced range between 159,646 bp and 160,178 bp in length. All the samples examined and 22 taxa re-annotated in *Sorbus *sensu lato (*Sorbus s.l.*) contain 113 unique genes with 19 of these duplicated in the inverted repeat (IR). Six hypervariable regions including *trnR*-*atpA*, *petN*-*psbM*, *rpl32-trnL*, *trnH*-*psbA*, *trnT*-*trnL* and *ndhC-trnV* were screened and 44–53 SSRs and 14–31 dispersed repeats were identified as potential molecular markers. Phylogenetic analyses under ML/BI indicated that *Sorbus s.l.* is polyphyletic, but *Sorbus s.s.* and the other five segregate genera, *Aria*, *Chamaemespilus*, *Cormus*, *Micromeles* and *Torminalis* are monophyletic. Two major clades and four sub-clades resolved with full-support within *Sorbus s.s*. are not consistent with the existing infrageneric classification. Two subgenera, subg. *Sorbus* and subg. *Albocarmesinae* are supported as monophyletic when *S. tianschanica* is transferred to subg. *Albocarmesinae* from subg. *Sorbus* and *S. hupehensis* var. *paucijuga* transferred to subg. *Sorbus* from subg. *Albocarmesinae*, respectively. The current classification at sectional level is not supported by analysis of cp genome phylogeny.

**Conclusion:**

Phylogenomic analyses of the cp genomes are useful for inferring phylogenetic relationships in *Sorbus s.s*. Though genome structure is highly conserved in the genus, hypervariable regions and repeat sequences used are the most promising molecule makers for population genetics, species delimitation and phylogenetic studies.

**Supplementary Information:**

The online version contains supplementary material available at 10.1186/s12870-022-03858-5.

## Introduction

The genus *Sorbus* L. (Maleae, Rosaceae), when established by Linnaeus [1], included only two pinnately leaved species, *S. aucuparia* L. and *S. domestica* L. The simple leaved species in *Sorbus *sensu lato (*Sorbus s.l.*) known to Linnaeus were assigned to other genera in the tribe Maleae. The taxonomy of *Sorbus* has historically been controversial. Taxonomists either adopted a broad definition [2–6] or segregated it into six small genera, i.e., *Aria* (Pers.) Host, *Chamaemespilus* Medik., *Cormus* Spach, *Micromeles* Decne., *Sorbus *sensu stricto (*Sorbus s.s.*) and *Torminalis* Medik., with varied delimitation [1, 7–12]. Evidence from morphological [11, 13] and molecular analyses [14–19] suggested that *Sorbus s.l.* is polyphyletic and can be divided into five or six separate evolutionary lineages. Accordingly, *Sorbus s.l.* has been divided into five or six genera, and the genus *Sorbus s.s.* is restricted to species with pinnately compound leaves and small fruits [12].

Currently, *Sorbus s.s.* consists of about 90 species with more than 60 native to China [5, 6, 12]. The genus is distributed in the temperate regions of the Northern Hemisphere with the greatest diversity in the mountains of south-western China, adjacent areas of Upper Burma and the Eastern Himalaya [12]. *Sorbus s.s.* species have great horticultural potential for their autumn leaf color and late summer and autumn fruit displays which range in colour from scarlet and deep crimson to orange, pink, yellow and pure white. However, relationships within the genus are still unresolved due to interspecific hybridization, apomictic polyploidy and the limited phylogenetic research data available, so intrageneric classifications proposed by previous taxonomists need to be tested. In the twentieth century, the broad definition of the *Sorbus* was adopted by most authors and the genus *Sorbus s.s.* was usually treated as a subgenus or a section within *Sorbus s.l.* Koehne [20] classified subg. *Aucuparia* (equivalent to *Sorbus s.s.*) into five unnamed groups because it was "impossible to divide the genus into well characterized sections". Yü and Kuan [21] separated sect. *Sorbus* (equivalent to *Sorbus s.s.*) into eight series based on morphological characters such as the presence of trichomes on the buds, the number and shape of the leaflets and the fruit color. Gabrielian [4] argued that some series proposed by Yü and Kuan [21] included distantly related taxa and assigned species of sect. *Sorbus* in Western Asia and the Himalayas to nine subsections based on comparative morphological and anatomical data. Phipps et al*.* [5] divided subg. *Sorbus* (equivalent to *Sorbus s.s.*) into two sections, nine series and five informal groups based on morphological characters such as the number and size of leaflets, free or united carpels and fruit color. The only recent revision of the genus S*orbus s.s.* was by published McAllister [12], who divided the genus into two subgenera and 11 sections based mainly on morphological characters such as the color of hairs on the buds, the number, size and shape of leaflets and the color of the fruits, combined with ploidy levels, breeding systems and geographical distribution. Both infrageneric classification and taxonomic inconsistency in species delimitation remain a challenge in the genus. The identities of *S. rehderiana* Koehne and *S. koehneana* C.K.Schneid. are examples discussed here. *S. hypoglauca* (Cardot) Hand.-Mazz. was treated as a synonym of *S. rehderiana* by Yü and Lu [3] and Lu and Spongberg [6], *S. unguiculata* Koehne was reduced to the synonymy of *S. koehneana* by McAllister [12], while both of them were recognized as distinct species by McAllister [12] and Phipps et al*.* [5].

Previous molecular studies mainly focused on the phylogeny of the tribe Maleae, while few concentrated specifically on the infrageneric relationships within *Sorbus s.s*. Despite previous efforts to elucidate infrageneric relationships within the genus, relationships between the subgenera and sections have remained uncertain. Phylogenetic analyses using chloroplast markers [16–18, 22] or chloroplast (cp) genomes [19] supported the monophyly of the genus but did not support any existing infrageneric classifications. However, conflicting results were noted in nuclear DNA phylogenies. Based on ITS, Wang and Zhang [23] suggested that *Sorbus s.l.* was monophyletic, but that *Sorbus s.s* and the infrageneric groups were not monophyletic based on ITS phylogeny. Contrary to Wang and Zhang [23], based on ITS, Li et al*.* [24] supported the monophyly of *Sorbus s.s.* and the other four segregated segregated genera from *Sorbus s.l.*, *i.e.*, *Aria* (including *Micromeles*), *Chamaemespilus*, *Cormus* and *Torminalis*.

Chloroplast genomes of most vascular plants range from 120 to 160 kb, and their cp genomes have a conserved quadripartite structure composed of two copies of an inverted repeat (IR) which divides the remainder of the genomes into one large and one small single copy regions (LSC and SSC) [25]. Chloroplast genomes are frequently used in systematics for the simplicity of the circular structure, predominantly clonal inheritance along the maternal line, as well as being highly variable even at low taxonomic levels [26]. Knowledge of the organization and evolution of cp genomes in *Sorbus s.s.*, *Sorbus s.l.* and tribe Maleae has been expanding rapidly because of the fast growing number of completely sequenced genomes available. Currently, the cp genomes of more than 100 species in the tribe Maleae including 22 species of *Sorbus s.l.* have been reported and are available for use (https://www.ncbi.nlm.nih.gov)*.*

Thus, in genus *Sorbus s.s.*, relationships among subgenera and sections remained uncertain. In this study, cp genomes of 15 *Sorbus s.s.* samples and an unidentified sample were newly sequenced and compared with other 22 other samples of *Sorbus s.l.* and 26 samples of other genera from tribe Maleae. The aims were: (1) to determine the structure of cp genomes in the 16 *Sorbus s.s.* samples; (2) to compare the structural variation, investigate and screen mutational hotspots, examine variations of simple sequence repeats (SSRs) and dispersed repeat sequences, and to calculate the nucleotide diversity in *Sorbus s.s.* cp genomes for future population genetic, species delimitation and phylogenetic studies; (3) to reconstruct phylogenetic relationships among species in *Sorbus s.s.* and *Sorbus s.l.*

## Results

### Organization and features of the chloroplast genomes

**Fig. 1:**
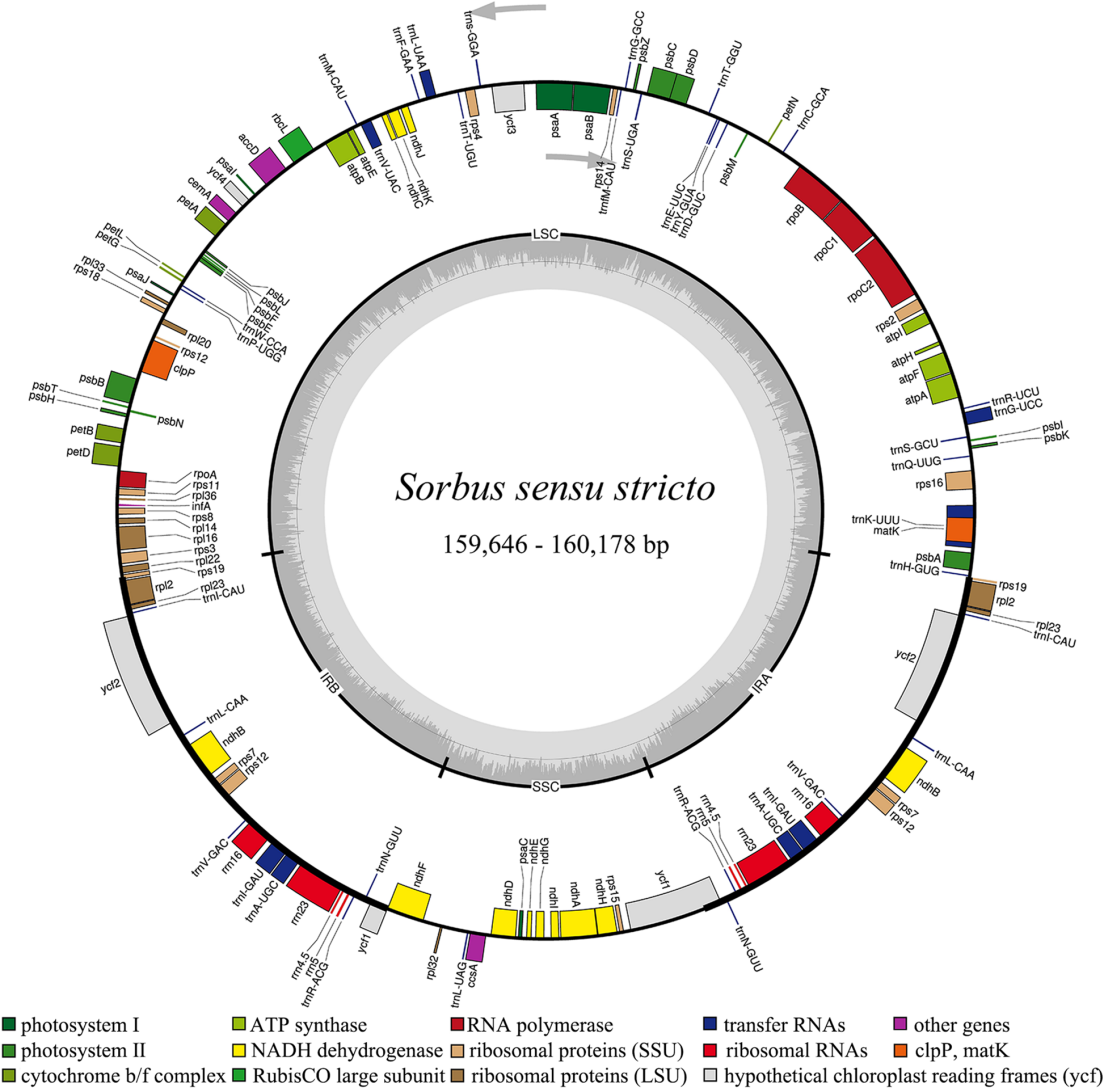
Gene map of 16 *Sorbus s.s.* chloroplast genomes. The outer circle shows the genes at each locus, and inverted repeat regions are indicated with thicker lines. Genes on the outside of the outer circle are transcribed in a counterclockwise direction, while genes on the inside of the outer circle are transcribed in a clockwise direction. The inner circle indicates the range of the LSC, SSC, and IRs, and also shows a GC content graph of the genome. In the GC content graph, the dark gray lines indicate GC content, while light gray lines indicate the AT content at each locus

The chloroplast genomes of the 15 species and the unidentified sample of *Sorbus s.s.* exhibit similar structure and organization (Table S1, Fig. 1). The size of the cp genomes of the 16 *Sorbus s.s.* samples range from 159,646 bp in *S. wilsoniana* C.K.Schneid. to 160,178 bp in *S. hypoglauca*. All the 16 cp genomes consist of a large single-copy (LSC) with lengths between 87,612 bp in *S. sargentiana* Koehne and 88,125 bp in *S. hypoglauca*, a small single-copy (SSC) with lengths between 19,219 bp in *Sorbus* sp. and 19,359 bp in *S. tianschanica* Rupr.; and a pair of inverted repeats (IRs) with lengths between 26,378 bp (*S. aestivalis* Koehne and the other nine taxa) and 26,405 bp (*S. amabilis* Cheng ex T.T.Yu; Table S1). The total GC content is nearly similar, 36.5% for five samples and 36.6% for the other 11 samples (Table S1).

All the 16 cp genomes assembled here encode 113 unique genes (79 protein-coding genes, 30 tRNA genes and four rRNA genes), and 19 of these are duplicated in the IR, giving a total of 132 genes (Tables S1, S2 and Fig. 1). Eighteen genes contain one (*atpF, ndhA*, *ndhB*, *petB*, *petD*, *rpl2*, *rpl16*, *rpoC1*, *rps12*, *rps16, trnA-UGC*, *trnG-UCC*, *trnI-GAU*, *trnK-UUU*, *trnL-UAA* and *trnV-UAC*) or two (*clpP* and *ycf3*) introns, and six of these are the tRNA genes (Table S2, Fig. 1). The cp genomes consist of 56.5 or 56.6% coding regions (49.1 or 49.2% protein coding genes and 7.4% RNA genes) and 43.4 or 43.5% non-coding regions, including both intergenic spacers and introns (Table S1).

**Fig. 2:**
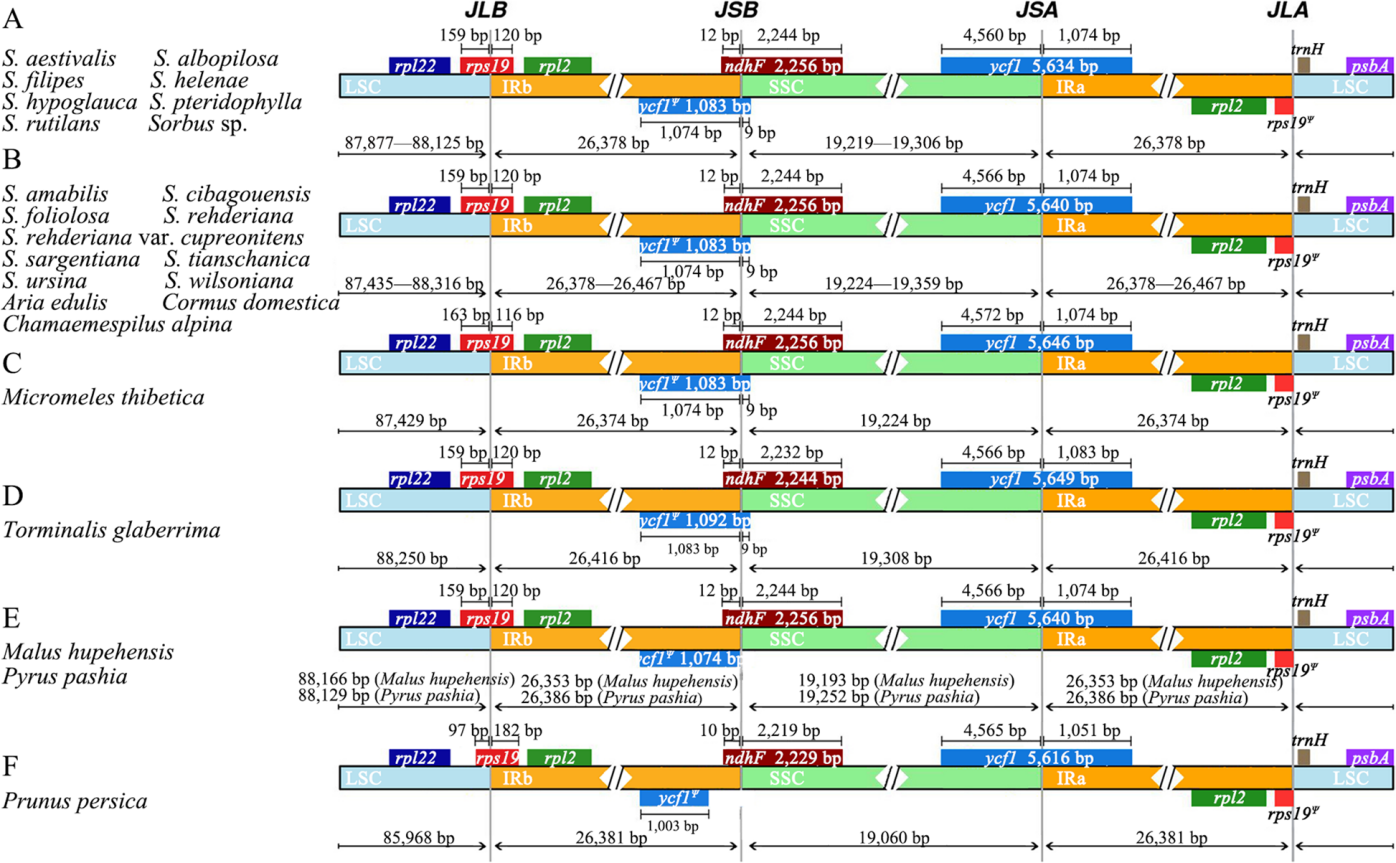
Comparisons of the LSC, SSC and IRs boundaries (**A**–**D**) within *Sorbus s.l.* and (**E** and **F**) among three other Rosaceae cp genomes. Genes shown below are transcribed forward and those shown above the lines are transcribed in reverse. Minimum and maximum sizes for the regions and structures of each chloroplast type that compose the borders are indicated in base pairs (bp)

The boundaries between the IR and LSC/SSC regions of the 16 *Sorbus s.s.* cp genomes and the eight species in other genera in Rosaceae were compared (Fig. 2). The IRb/LSC boundary is located within the *rps19* gene (the 5′ end of the *rps19* is located in the IRb region while 3′ end is located in the LSC), therefore creating a pseudogene of the 5′ end of this gene (*rps19*^*Ψ*^) in the IRa region in all cp genomes compared. The length of *rps19*^*Ψ*^ is 116 bp in *Micromeles thibetica* (Cardot) Mezhenskyj (Fig. 2 C), 182 bp in *Prunus persica* (L.) Batsch (Fig. 2 F), and 120 bp in the other 22 species (Fig. 2 A–B, D, E). The IRa/LSC border is adjacent to the *rps19*^*Ψ*^ in all species except in *Micromeles thibetica* (Fig. 2 C), which is within the *rps19*^*Ψ*^. The IRa/SSC boundary is located in the *ycf1* gene (the 5′ end of the *ycf1* is located in the IRa region while the 3′ end is located in the SSC), thus creating a pseudogene of the 5′ end of this gene (*ycf1*^*Ψ*^) in the IRb region. The size of *ycf1*^*Ψ*^ range from 1,003 (*Prunus persica*; Fig. 2 F) to 1,092 bp (*Torminalis glaberrima* (Gand.) Sennikov & Kurtto; Fig. 2 D), and 1,083 bp in all the *Sorbus s.s.* species (Fig. 2 A–C, E). The IRb/SSC boundary slightly varies: 21 species are located within the overlapping region of the pseudogene *ycf1*^*Ψ*^ and *ndhF*, while the other three species (*Malus hupehensis* (Pamp.) Rehder, *Prunus persica*, *Pyrus pashia* Buch.-Ham. ex D.Don) are located within the *ndhF* gene (Fig. 2 E, F).

### Codon preference analysis

According to the codon usage analysis, the total sequence sizes of the protein coding genes are 78,570–78,588 bp in the 16 *Sorbus s.s.* genomes; 26,190–26,196 codons were encoded (Table S3). Leucine encoded with the highest number of codons ranging from 2,753 to 2,757, followed by isoleucine, with the number of codons encoded between 2,255 and 2,260. Cysteine is the least (297 or 298)*.* The relative synonymous codon usage (RSCU) values vary a little among the 16 *Sorbus s.s.* sequences. Thirty codons are used frequently with RSCU > 1 and 32 codons used less frequently with RSCU < 1. UUA shows a preference in all the 16 cp genomes. The frequency of use for the start codons AUG and UGG, encoding methionine and tryptophan, show no bias (RSCU = 1). Codons with A (32.1%) or U (38.2%) in the third position are all 70.3%, thus the codon usage is biased towards A or U at the third codon position.

### Repeated sequences analysis

**Fig. 3:**
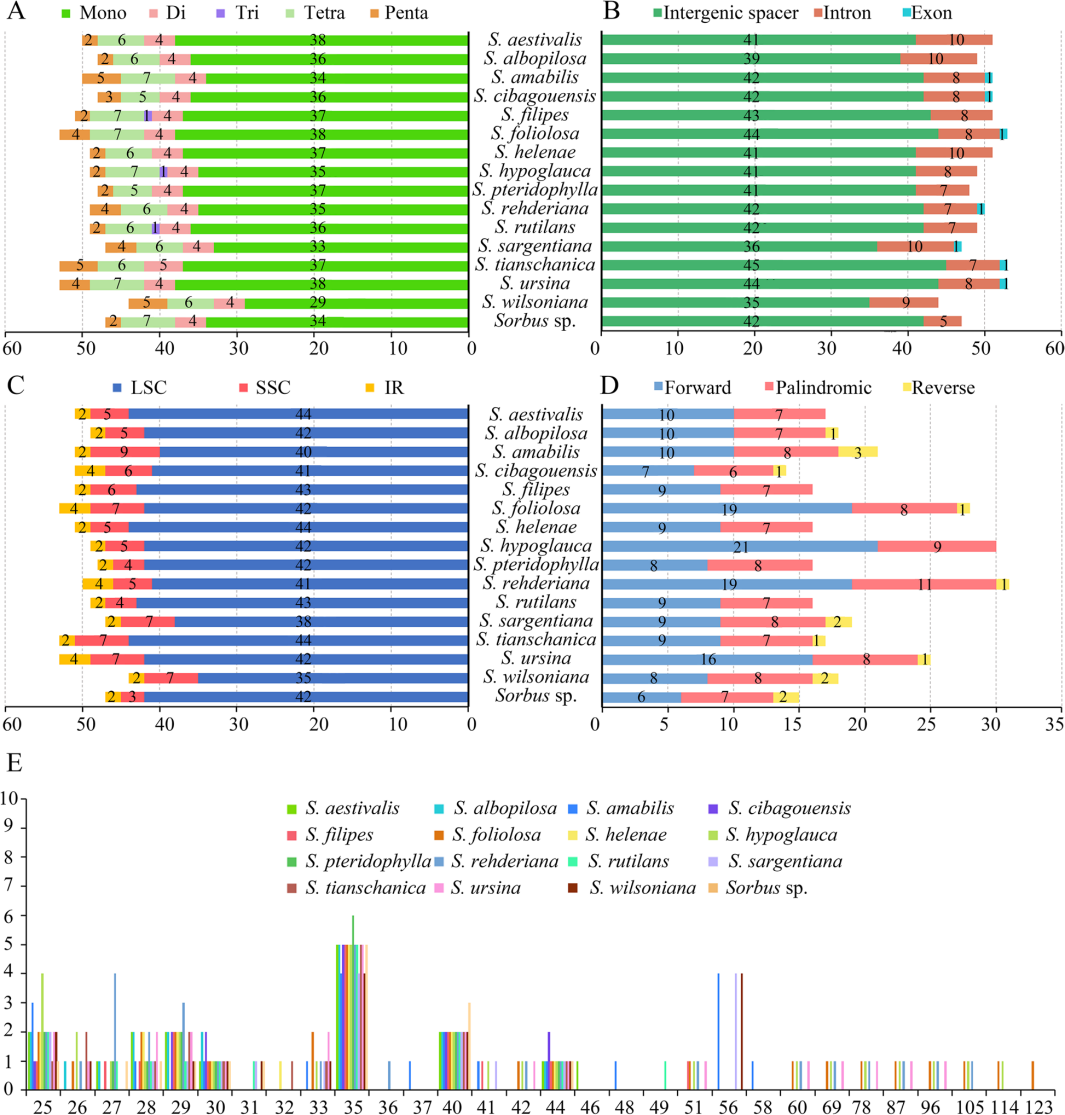
Distribution of repeats in 16 *Sorbus s.s.* samples. **A** Distribution of SSRs types. **B** Distribution of SSRs among intergenic spacer, intron and exon regions. **C** Distribution of SSRs in LSC, SSC and IR regions. **D** Type of forward, reverse, palindromic and complement repeats. **E** Frequency of forward, reverse, palindromic and complement repeats

The total number of SSRs in 16 *Sorbus s.s.* genomes ranges from 44 to 53 (Fig. 3 A–C; Table S4). The most abundant SSRs are A or T nucleotide repeats, which account for 88.2 to 96.3% (Table S4) of the total. The most common SSRs are mononucleotides, which range from 29 to 38, followed by tetranucleotides ranging from 5 to 7, and pentanucleotides ranging from 2 to 5. Dinucleotides are all four in the examined samples except for five in *S. tianschanica*. Trinucleotides were discovered only in three species: *S. filipes* Hand.-Mazz., *S. hypoglauca* and *S. rutilans* McAll. There are three hexanucleotides in *S. cibagouensis* H.Peng & Z.J.Yin, two in *S. helenae* Koehne, one in *S. aestivalis*, *S. albopilosa* T.T.Yu & L.T.Lu, *S. amabilis* and *S. rehderiana*, and none in the other 11 samples. SSRs are mainly distributed in the intergenic regions (76.6–89.4%), with much lower quantities distributed in the intron regions (10.6–21.3%) and exon regions (0–2.1%; Fig. 3B). Furthermore, SSRs are found mainly in LSC regions (78.4–89.4%), and are significantly lower in the SSC (6.4–17.6%) and IR (3.8–8%) regions (Fig. 3 C)*.*

The REPuter screening discovered 14 to 31 dispersed repeats of 25 bp or longer among the 16 *Sorbus s.s.* cp genomes examined (Fig. 3 D–E). *Sorbus rehderiana* has the largest number of repeats with 31 and *S. cibagouensis* has the fewest with only 14. The majority of the repeats (19.1–44.4%) in all cp genomes range between 25 and 29 bp. The longest repeats is 123 bp and was only found in *S. foliolosa* Spach. Six taxa, *S. albopilosa*, *S. cibagouensis*, *S. helenae*, *S. pteridophyslla* Hand.-Mazz., *S. tianschanica* and *Sorbus* sp., have a maximum size of 44 bp. Only four taxa, *S. foliolosa*, *S. hypoglauca*, *S. rehderiana* and *S. ursina* S.Schauer, have repeats with a size larger than 60 bp (Table S5, Fig. 3 E). Among these, forward repeats (6–21) were the most common, followed by palindromic repeats (6–11) and reverse repeats (1–3, Fig. 3 D).

### Comparative analysis of chloroplast genomes

**Fig. 4:**
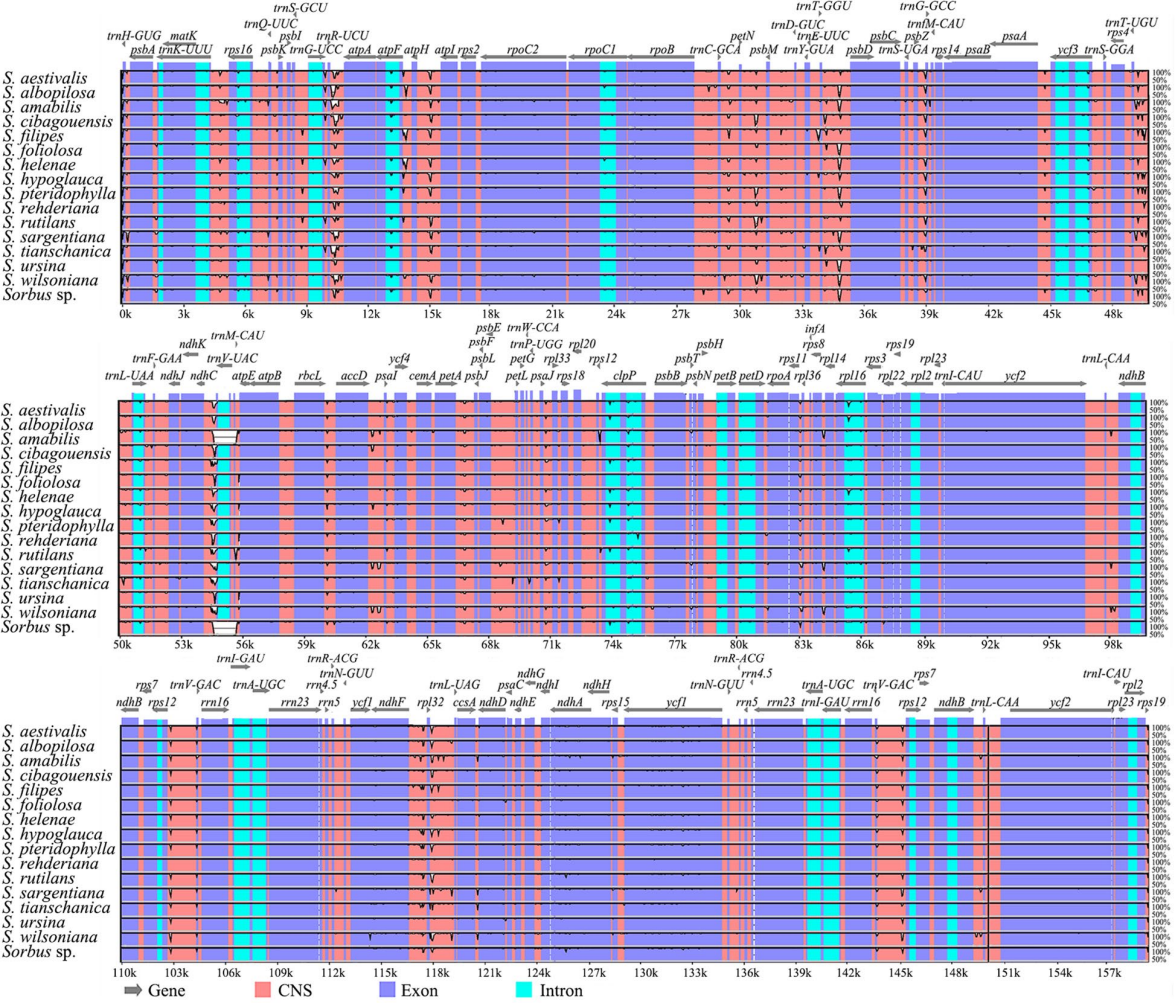
Comparison of 16 assembled *Sorbus s.s.* chloroplast genomes using mVISTA. Complete cp genomes of *Sorbus s.s.* samples were compared using *S. insignis* Hedl. as a reference. Purple blocks indicate conserved genes, while red blocks indicate noncoding sequences (CNS). White blocks represent regions with sequence variation among the 16 *Sorbus s.s.* samples. Gray arrows indicate the direction of gene transcription

Comparative cp genome analysis reveals that noncoding regions are generally more divergent than coding regions and LSC/SSC regions are more divergent than IR regions (Fig. 4). The highest levels of divergence were found in 17 intergenic regions, 15 in the LSC regions, namely *trnH-psbA, trnK-rps16*, *trnG-trnR*, *trnR-atpA*, *atpF-atpH*, *atpH-atpI*, *trnC-petN*, *petN-psbM*, *trnT-psbD*, *psbZ-trnG*, *trnT-trnL*, *ndhC-trnV, trnM-atpE, accD-psaI* and *rps8-rpl14*; and two in the SSC regions, namely *ndhF-rpl32, rpl32-trnL*. Apart from these regions, two intron regions: *clpP* and *rpl16* also show high sequence variation.

**Fig. 5:**
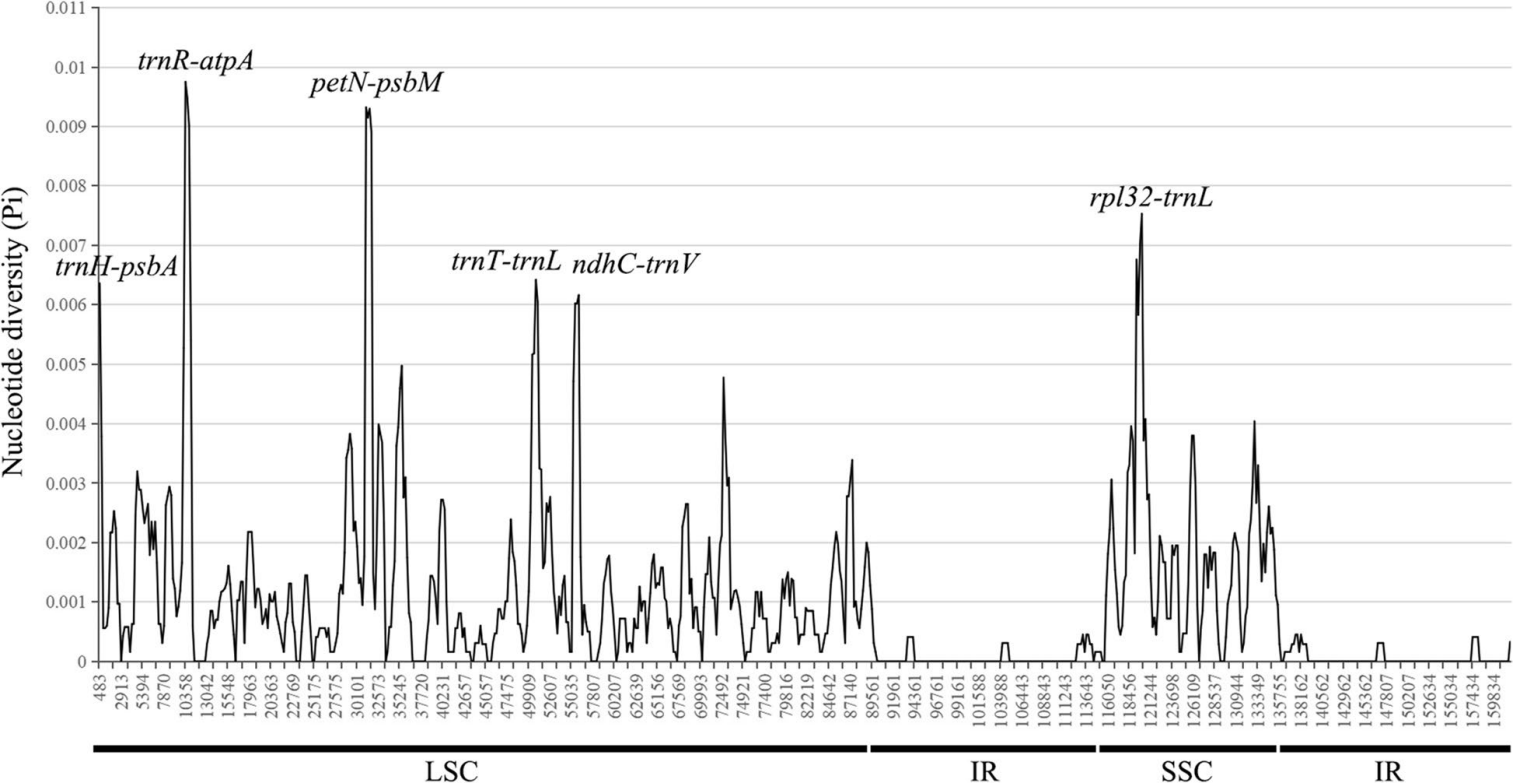
Sliding window analysis of 16 *Sorbus s.s.* cp genome alignment. Window length: 800 bp; step size: 200 bp. X-axis: position of the midpoint; Y-axis: nucleotide diversity (Pi)

To elucidate levels of diversity at the sequence level, the nucleotide diversity (Pi) values were calculated. The Pi values range from 0 to 0.00975, with mean value of 0.00098 (Fig. 5, Table S6). The SSC region shows the highest nucleotide diversity (Pi = 0.00173), while the lowest Pi is in the IR boundary regions (Pi = 0.00016). Meanwhile, six hypervariable sites with Pi between 0.005 and 0.01 were screened, which are *trnR*-*atpA* (Pi = 0.00975), *petN*-*psbM* (Pi = 0.00932), *rpl32-trnL* (Pi = 0.00753), *trnH*-*psbA* (Pi = 0.00636), *trnT*-*trnL* (Pi = 0.00642) and *ndhC-trnV* (Pi = 0.00616).

### Phylogenetic analysis

**Fig. 6:**
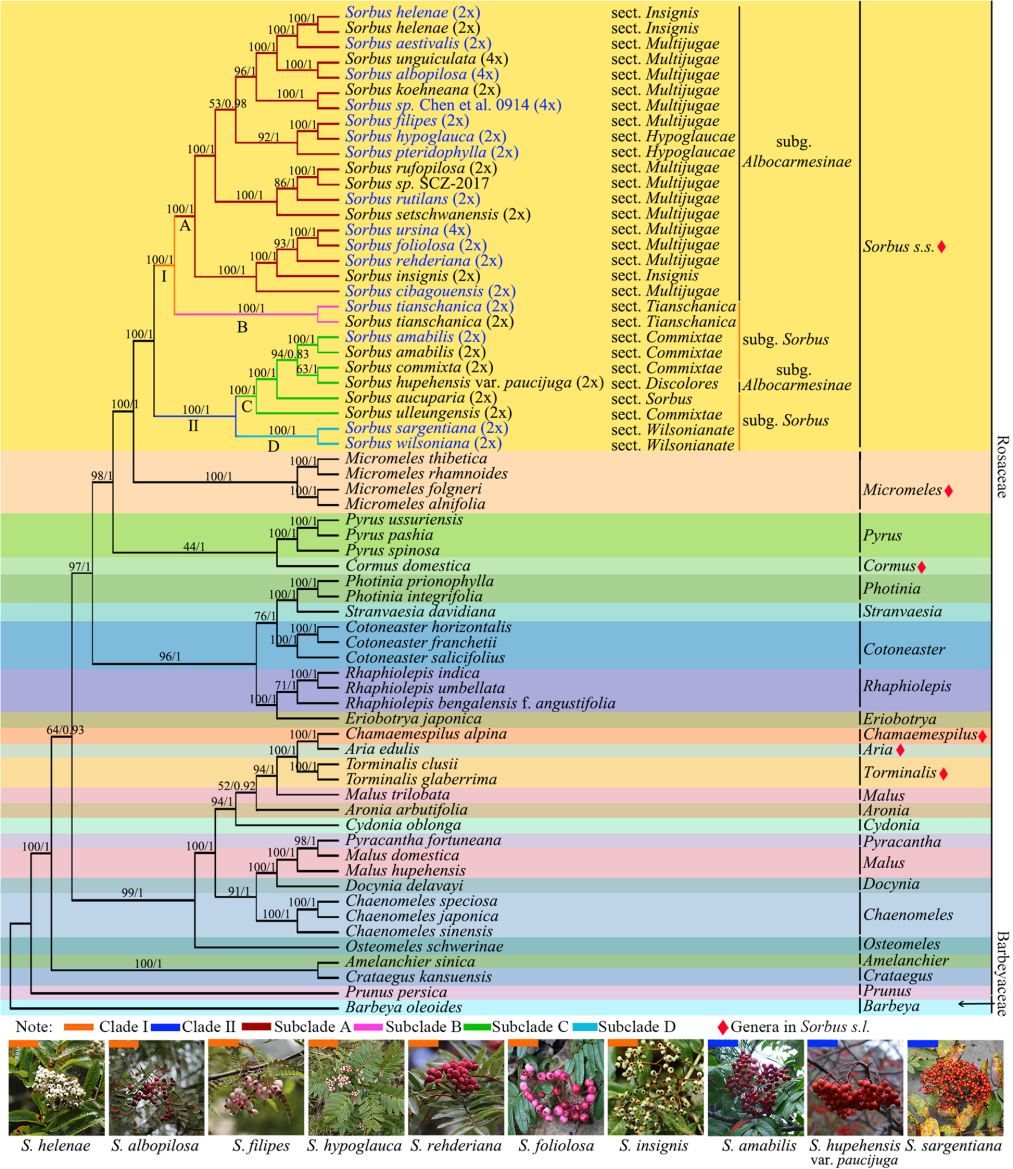
Phylogenetic tree based on complete cp genomes resulting from the maximum likelihood (ML) analysis and Bayesian inference (BI) analysis. Bootstrap values in ML analysis and posterior probabilities in BI analysis are listed at nodes. Names of taxa newly sequenced in *Sorbus s.s.* are in blue

The ML and BI analyses of cp genomes result in highly congruent topologies. There are only slight differences in support values between the phylogenetic trees. Therefore, only the ML topology is shown here with the ML/BI support values added at each node (Fig. 6).

Our analyses confirmed that *Sorbus s.l.* is polyphyletic and the six segregate genera, *i.e.*, *Aria*, *Chamaemespilus*, *Cormus*, *Miromeles*, *Sorbus s.s.* and *Torminalis*, are monophyletic. *Aria*, *Chamaemespilus* and *Torminalis* are resolved in one branch near the base of the cp genome phylogeny together with *Malus trilobata* C.K. Schneid., *Aronia arbutifolia* (L.) Pers. and *Cydonia oblonga* Mill. *Miromeles* is sister to *Sorbus s.s.* and nested in one branch with *Cormus* and *Pyrus* L*.*

Within the monophyletic genus *Sorbus s.s.*, two major clades are resolved. Clade I comprises two fully supported subclades (A and B). Subclade A is consistent with subg. *Albocarmesinae* McAll. Nevertheless, three sections, sect. *Hypoglaucae* McAll., sect. *Insignes* (T.T. Yu) McAll. and sect. *Multijugae* (T.T.Yu) McAll. within subg. *Albocarmesinae*, are not monophyletic. Subclade B contains two samples representing *S. tianschanica* which belongs to subg. *Sorbus* sect. *Tianshanicae* (Kom. ex T.T.Yu) McAll., although it is resolved with full-support on a branch with subg. *Albocarmesinae* with full-support. Clade II contains two fully supported subclades (C and D) and is sister to the rest of the genus. Subclade C includes five taxa belonging to three different sections from two subgenera. *Sorbus aucuparia* in sect. *Sorbus* McAll. (subg. *Sorbus*) and *S. hupehensis* var. *paucijuga* in sect. *Discolores* (T.T.Yu) McAll. (subg. *Albocarmesinae*), are nested within sect. *Commixtae* McAll. (subg. *Sorbus*)*.* Subclade D contains two species in subg. *Sorbus* sect. *Wilsonianae* McAll.

## Discussion

### Gene, structure and the potential molecular markers

*Sorbus s.s.* can be easily identified by the pinnate leaves and colorful fruits with persistent sepals, stamens and styles. Understanding of the taxonomy and phylogenetic relationships in *Sorbus s.s.* has been particularly difficult because of the widespread occurrence of polyploidy associated with gametophytic apomixis [27–29]. In the present study, 29 *Sorbus s.s.* cp genomes (16 newly sequenced and 13 previously reported) representing 23 species, one variety and two unidentified taxa from both subgenera and eight out of the 11 sections were compared in all cp genomes in *Sorbus s.l.* to clarify phylogenetic relationships and resolve taxonomic uncertainties.

The structure, gene order and GC content are highly conserved and nearly similar in *Sorbus s.s.* samples analyzed here, and are identical to other cp genomes in angiosperms [30–35]. The size of the 29 cp genomes varied from 159,632 (*S. ulleungensi*s Chin S. Chang; NC037022) to 160,178 bp (*S. hypoglauca*). The *Sorbus s.s.* cp genomes sequenced here all contained 113 unique genes with the total GC content being 36.5 or 36.6% (Table S1). However, the absence of one to six of the following genes: *infA*, *psaC*, *psbL*, *rpl16*, *rrn4.5*, *rrn5*, *rrn16*, *rrn23*, *trnG-GCC*, *trnG-UCC*, *trnI-CAU* and *trnS-GGA*, were reported in 22 species in *Sorbus s.l*. (https://www.ncbi.nlm.nih.gov/, Table S8). Some species were found to contain different numbers of genes in different individuals, for examples, *S. amabilis* (MT357029) and *S*. *helene* (KY419924) were reported to contain 109 and 111 genes respectively, but both annotated 113 genes in the samples examined here. To eliminate the influence of annotation software and references used, the 22 samples were all re-annotated using Plastid Genome Annotator (PGA) program [36] and Geneious v.9.0.2 [37] with *S. insignis* (NC051947) and *Malus hupehensis* (NC040170) as references. Unexpectedly, no gene loss was found and all the 22 sequences re-annotated contained 113 genes which were identical to the samples examined in this study (Table S8).

Genome composition and natural selection are the two major factors affecting codon usage bias [38, 39]. The total number of 64 codons present across the *Sorbus s.s.* cp genomes encoding 20 amino acids (AAs) and codon usage is biased towards A or U at the third codon position, which is in consistent with other higher plants [40–43].

The contraction and expansion of IR regions are useful in evolutionary studies in some taxa [44]. However, the IR/SC boundaries are conserved in *Sorbus s.s.* and in most species of *Sorbus s.l.* All species compared in this study with the IRb/LSC boundary were located within the *rps19* gene and creating a pseudogene (*rps19*^*Ψ*^) in the IRa, the IRa/SSC boundary located in the *ycf1* gene and creating a pseudogene (*ycf1*^*Ψ*^) in the IRb region.

SSRs are useful markers to assess the organization of genomes and diversity at the species and population levels [45–47] and to analyse phylogenetic relationships in plants [48]. In this study, the number of SSRs found within *Sorbus s.s.* genomes ranges from 44 to 53, similar to SSRs previously documented in the genus [49, 50]. Consistent with the previous reports in other *Sorbus s.s.* species, mononucleotides are the most common SSRs and the largest amount of SSRs is located in the intergenic regions. SSRs are especially useful in establishing the amount of genetic diversity within and between populations [51] and in investigating the parentage of polyploid in *Sorbus s.s.* [52]. Dispersed repetitive sequences represent a major component of genomes and play a major role in genomic rearrangement and sequence variation [53, 54]. *Sorbus s.s.* species contain a substantial number of dispersed repeats and show marked difference in number ranging from 58 to 130 with a majority of the repeats ranging from between 20 and 25 bp.

Despite the high levels of gene conservation observed, 17 intergenic regions and two introns genes were identified as highly divergent in *Sorbus s.s.* (Figs. 4 and 5). Among them, some were shown in previous studies to be highly variable and of high phylogenetic utility, such as *trnK-rps16*, *atpH-atpI*, *trnT-psbD*, *ndhC-trnV*, *ndhF-rpl32* and *rpl32-trnL* [34, 55]. Consistent with the diverse patterns found in most angiosperms [56–58], sequence divergence in non-coding regions is higher than that in coding regions. Variable chloroplast sequences have been widely used for plant phylogeny reconstruction [58, 59] and for species identification [60, 61]. However, among the chloroplast sequences which have most frequently been used in phylogeny reconstruction of tribe Maleae, such as *trnH-psbA*, *trnL*-*trnF* and *trnG-trnS, *etc*.* [15, 17, 18], only one intergenic region, *trnH-psbA* (Pi = 0.00339; ranked 5) and one intron *rpl16* (Pi = 0.00339; ranked 15) show high variability in *Sorbus s.s.* The only one chloroplast marker, *rps16-trnK*, which was applied in the phylogeny and historical biogeography analysis of *Sorbus s.s* [22]*.* with Pi value (0.0032) ranked 16. Furthermore, the intergenic region *trnR-atpA* shows the highest Pi value (0.00975) in all *Sorbus s.s.* genomes, and is also hypervariable within the genomes of other species in Rosaceae genomes [18, 33, 62]. Rapid evolutionary rates of the six hypervariable regions, *trnR*-*atpA*, *petN*-*psbM*, *rpl32*-*trnL*, *trnT*-*trnL*, *trnH*-*psbA* and *ndhC*-*trnV*, have led to some topology confusion (Figs. S1,S2,S3,S4,S5,S6, and S7). Howerver, the phylogenetic analysis based on *trnR*-*atpA* and *trnT*-*trnL* resulted in similar topology as the complete cp genomes (Figs. S1, and S4), *rpl32*-*trnL* and *trnT*-*trnL* could well differentiated the two subgenera in *Sorbus s.s.* (Figs. S3, and S4). Meanwhile, *rpl32*-*trnL*, *trnT*-*trnL* and the combination performed well in species identification which grouped the two individuals of same species included in the studied together (Figs. S3, and S4). Thus, the new highly variable sequences generated in this study, especially the six hypervariable regions, *trnR-atpA*, *petN-psbM*, *rpl32-trnL*, *trnT-trnL*, *trnH-psbA* and *ndhC-trnV*, might be the most promising potential molecule makers in phylogeny reconstruction and DNA barcode identification for *Sorbus s.s.* plants.

### Phylogenetic analysis

Chloroplast genomes are effective in inferring phylogenetic relationships at various taxonomic levels for the conservatism and uniparental heritance [63–65]. In this phylogenetic analysis using cp genomes, the monophyly and the infrageneric classification of *Sorbus s.s.* were investigated, as well as its relationship with other genera in Maleae. The status of *S. hypoglauca* and *S. unguiculata* was also re-evaluated.

In congruence with previous molecular phylogenetic studies [15, 17–19] and morphological research [11, 13], the generic circumscription of *Sorbus s.l.* is not supported by the phylogenetic analyses of this study. Six monophyletic lineages correspond to the six genera segregated from *Sorbus s.l.*, *Aria*, *Chamaemespilus*, *Cormus*, *Miromeles*, *Sorbus s.s.* and *Torminalis*, and are all well supported. However, the delimitations of three genera with simple leaves, *i.e.*, *Aria*, *Chamaemespilus* and *Micromeles*, was controversial. *Aria* was usually accepted in a broad sense in earlier morphological studies to include *Chamaemespilus* and *Micromeles* [11, 66, 67] or in molecular studies to include only *Micromeles* [14, 15, 24]. Our analyses indicated that Asiatic species formerly included in *Aria* with a persistent calyx are nested within *Micromeles* which forms the sister group of *Sorbus s.s*., but are only distantly related to *Aria edulis*, the type species of *Aria*. Therefore, it is proposed to treat *Micromeles* as an independent genus. All Asiatic simple leaved species formerly included in *Aria* have been transferred to *Micromeles* by Mezhenska et al*.* [68]. In our study, *Chamaemespilus* forms a sister group to *Aria*. The relationship between them needs to be further investigated.

The systematics of *Sorbus s.s.* have been discussed in terms of morphological [4, 5, 12, 20, 21] and molecular [17, 22–24] results. The topologies of the phylogenetic trees obtained here are congruent with that reported by Li et al*.* [22] using four nuclear markers (LEAFY-2, GBSSI-1, SBEI and WD) and one chloroplast marker (*rps16*-*trnK*). Corresponding to Li et al*.* [22], the two monophyletic clades resolved are largely congruent with the two subgenera, subg. *Albocarmesinae* and subg. *Sorbus*, as defined by McAllister [12]. However, the sections defined by McAllister [12] and infrageneric classification proposed by Koehne [20], Yü & Kuan [21], Gabrielian [4] and Phipps et al*.* [5], are not supported.

Species in clade I have white to crimson flowers, pinkish-red, white to pink or crimson fruits which will gradually become almost pure white with only the occasional crimson or pink fleck when ripe. Two monophyletic subclades, namely subclade A and subclade B are resolved in this clade. Subclade A consists of 16 species in subg. *Albocarmesinae* and two unidentified samples that are morphologically similar to species in this subgenus. Two species, *S. helenae* and *S. insignis* of sect. *Insignes* and two species of sect. *Hypoglaucae*, *S. hypoglauca* and *S. pteridophylla,* are nested within sect. *Multijug*ae. Thus, the three sections in subclade A, sect. *Hypoglaucae*, sect. *Insignes* and sect. *Multijug*ae are not monophyletic. McAllister [12] distinguished the three sections by the color of the hairs on buds and young shoots, whether petiole bases were sheathing or not and carpel apices free or fused, together with the ploidy levels. However, the two fully supported groups in subclade A lack of a consistent morphological synapomophy. Species in subclade A are sexual diploids or apomictic tetraploids. Four taxa, *S. albopilosa* (2C = 2.624 ± 0.047 pg), *S. unguiculata* (2C = 2.783 ± 0.103 pg), *S. ursina* (2C = 2.681 ± 0.028 pg) and the unidentified *Sorbus* sp. Chen et al*.* 0914 (2C = 2.765 ± 0.248 pg) are tetraploids (Chen et al. unpublished), the other 13 species are diploids [12, 69, 70]. The ploidy level of *Sorbu*s sp. SCZ-2017 is unknown. The tetraploids species, *S. albopilosa* and *S. unguiculata,* are clustered together and form a fully supported group with the diploid species *S. helenae* and *S. aestivalis*, *S. ursina* is grouped with the diploid species *S. foliolosa* and *Sorbus* sp. Chen et al*.* 0914 is grouped with diploid *S. koehneana*. However, the origin of tetraploid taxa and the relationship with the closely related diploid ones need further study. Subclade B contains two samples of *S. tianschanica,* a species that was formerly included in sect. *Tianshanicae* under subg. *Sorbus* by McAllister [12] and it is also a sexual diploid. In accordance with previous publications [22], *S. tianschanica* is sister to the sampled species of subg. *Albocarmesinae* and it suggests that the species may be misplaced. *Sorbus tianschanica* can be distinguished from all other species of subg. *Sorbus* by its “very glossy twigs” [12]. Furthermore, McAllister [12] noted that sect. *Tianshanicae* has fruits with a distinctive pinkish-red color unknown in subg. *Sorbus*, and he thought that it might indicate some relationship with species in subg. *Albocarmesinae*. Our results suggest the transfer of *S. tianschanica* to subg. *Albocarmesinae* from subg. *Sorbus*. However, more samples from other species of sect. *Tianshanicae* need to be sequenced to confirm its placement at sectional level.

Species in clade II could be easily distinguished from species in clade I by their white flowers and orange-red to bright red fruits which lack any trace of white or crimson [12, 24, 71]. All species in clade II are sexual diploids. In clade II, two subclades, subclades C and D, are fully supported. Morphologically, species in subclades C have much small inflorescences and relatively larger fruits compared to species in subclades D. Subclade C contains five taxa formerly assigned to three sections, sect. *Commixtae*, sect. *Sorbus* and sect. *Discolores*. Two taxa, *Sorbus aucuparia* of sect. *Sorbus* and *S. hupehensis* var. *paucijuga* of sect. *Discolores*, are nested within sect. *Commixtae*. Morphologically, *S. aucuparia* can be easily distinguished from the other two species in sect. *Sorbus*, *S. esserteauiana* Koehne and *S. scalaris* Koehne by its smaller stipules while the other two have larger, persistent stipules; *S. hupehensis* var. *paucijuga* is more closely related to *S. amabilis* and *S. commixta* in having white flowers and small red fruits rather than to *S. hupehensis* C.K. Schneid. which has white fruits. Therefore, *S. aucuparia* and *S. hupehensis* var. *paucijuga* might merit transfer to sect. *Commixtae*. Subclade D includes two species, *S. sargentiana* and *S. wilsoniana* of sect. *Wilsonianae* in subg. *Sorbus*, and it is the only one section whose monophyly is supported in the present study.

Taxonomic inconsistencies in species delimitation also remain a challenge in the genus *Sorbus s.s*. *S. hypoglauca* (Cardot) Hand.-Mazz. was treated as a synonym of *S. rehderiana* by Yü and Lu [3], and Lu and Spongberg [6], but it was reinstated as a distinct species by McAllister [12]. In the present study, *S. hypoglauca* is sister to *S. filipes* but not *S. rehderiana*. *Sorbus hypoglauca* differs from both *S. filipes* and *S. rehderiana* in having large persistent stipules. Therefore, there is good support for the treatment of *S. hypoglauca* as a distinct species. *S. unguiculata* Koehne was reduced to synonymy with *S. koehneana* by McAllister [12], but was treated as a distinct species by Phipps et al*.* [5]. In our study, *S. unguiculata* is not clustered with *S. koehneana*, but formed a sister group to *S. albopilosa*. Morphologically, *S. unguiculata* differs from *S. koehneana* by the much more numerous of leaflets, and from *S. albopilosa* by the white, not red fruits. Therefore, *S. unguiculata* might merit treatment as a distinct species.

## Conclusion

Complete chloroplast genomes of 29 samples in *Sorbus s.s.,* including 16 newly sequenced samples, representing both two subgenera and eight sections were compared. Although genome structure, organization and gene content are highly conserved in the genus, differences in number and distribution of repeat sequences and the six hypervariable regions could be used for molecular systematic, phylogeographic, and population genetic studies.

*Sorbus s.s.* and the other five genera segregated from *Sorbus s.l*. (*i.e.*, *Aria*, *Chamaemespilus*, *Cormus*, *Miromeles* and *Torminalis*) are strongly supported as monophyletic, while *Sorbus s.l.* is confirmed to be polyphyletic. The two subgenera of *Sorbus s.s.*, subg. *Sorbus* and subg. *Albocarmesinae* as defined by McAllister [12] are monophyletic when *S. tianschanica* is transferred to subg. *Albocarmesinae* and *S. hupehensis* var. *paucijuga* to subg. *Sorbus*. Nevertheless, except for sect. *Wilsonianae*, the seven sections in the genus *Sorbus s.s* as defined by McAllister [12] are not supported. To fully resolve relationships in *Sorbus s.s*., more cp genomes need be sequenced and phylogenetic analysis with cp genome and nrDNA data combined with morphological comparisons are necessary.

## Methods

### Sampling, DNA extraction and sequencing

Leaf samples representing 15 *Sorbus s.s.* species and an unidentified sample were collected in the field between 2015 and 2018 from Anhui, Hubei, Sichuan, Xinjiang, Xizang and Yunnan Provinces in China. Fresh leaves were rapidly dried using silica gel for further DNA extraction. Voucher specimens were deposited in the Herbarium of Nanjing Forestry University (NF) and collection information is listed in Table 1.

**Table 1:** Basic information of 16 *Sorbus s.s.* samples

Taxa	Voucher	Clean bases (G)	Clean reads	Average coverage ( ×)	Genbank accession number
*S. aestivalis*	CHINA. Sichuan, 29°39′42.46"N, 102°57′03.20"E, 2382 m, Xin Chen and Zhongren Xiong 0750 (NF)	2.35	7,200,013	46.6	ON049656
*S. albopilosa*	CHINA. Xizang, 29°49′22.06"N, 94°44′21.38"E, 3148 m, Xin Chen, Xiaochen Zhang and Zhongren Xiong 1030 (NF)	2.33	7,150,804	27.95	ON049662
*S. amabilis*	CHINA. Anhui, 30°08′09.60"N, 118°09′31.68"E, 1771 m, Xin Chen and Jiabao Li 1423 (NF)	4.52	13,851,710	62.15	ON049665
*S. cibagouensis*	CHINA. Xizang, 29°49′16.49"N, 95°42′40.63"E, 3272 m, Xin Chen, Xiaochen Zhang and Zhongren Xiong 0964 (NF)	2.90	8,895,466	61.75	ON049660
*S. filipes*	CHINA. Xizang, 29°48′23.40"N, 95°41′53.27"E; 3598 m, Xin Chen, Xiaochen Zhang and Zhongren Xiong 0988 (NF)	4.32	13,335,748	72.05	ON049661
*S. foliolosa*	CHINA. Xizang, 29°29′24.94"N, 94°55′14.81"E, 3650 m, Xin Chen 0620 (NF)	3.53	10,877,503	87.1	ON049652
*S. helenae*	CHINA. Sichuan, 29°31′26.41"N,103°20′12.88"E, 3014 m, Xin Chen, Xiaoyan Wang and Chunhui Wang 1937 (NF)	2.60	8,333,965	205.2	ON049667
*S. hypoglauca*	CHINA. Yunnan, 25°41′14.47"N, 100°06′32.61"E, 3211 m, Xin Chen, Xiaochen Zhang and Qi qi 1237 (NF)	2.36	7,244,523	12.8	ON049664
*S. pteridophylla*	CHINA. Xizang, 29°43′32.18"N, 95°38′41.52"E, 3224 m, Xin Chen 0572 (NF)	2.32	7,128,300	45.45	ON049651
*S. rehderiana*	CHINA. Sichuan, 30°01′21.08"N, 100°49′38.46"E, 4305 m, Xin Chen 0556 (NF)	2.34	7,192,370	32.95	ON049650
*S. rutilans*	CHINA. Sichuan, 29°59′20.01"N, 102°01′96.00"E, 3060 m, Xin Chen and Zhongren Xiong 0739 (NF)	2.90	8,905,432	155.35	ON049654
*S. sargentiana*	CHINA. Sichuan, 29°39′43.21"N, 102°57′03.97"E, 2387 m, Xin Chen and Zhongren Xiong 0749 (NF)	4.92	15,204,888	28.85	ON049655
*S. tianschanica*	CHINA. Xinjiang, 43°52′52.76"N, 88°07′44.44"E, 1927 m, Wenhao Fan 1761 (NF)	2.58	8,291,047	238.9	ON049666
*S. ursina*	CHINA. Sichuan, 30°53′35.86"N, 102°58′37.91"E, 3411 m, Xin Chen and Zhongren Xiong 0664 (NF)	3.22	9,861,892	97.2	ON049653
*S. wilsoniana*	CHINA. Hubei, 30°01′34.84"N, 109°45′42.59"E, 1856 m, Xin Chen and Yun Chen 0784 (NF)	3.45	10,636,280	58.75	ON049657
*Sorbus* sp.	CHINA. Xizang, 29°31′27.88"N, 96°46′43.05"E, 4146 m, Xin Chen, Xiaochen Zhang and Zhongren Xiong 0914 (NF)	2.91	8,920,370	110.75	ON049659

Total DNA was extracted following the CTAB protocol [72]. DNA was quantified through fluorometry using Qubit Fluorometer or microplate reader and visualized in a 1% agarose-gel electrophoresis for quality check. The extracted genomic DNA was subjected to random degradation by Covaris, and then fragments with a size of 270 bp were selected by using AxyPrep Mag PCR clean up Kit. The selected fragments were amplified after suffering from end repair, the addition of polyA tail and adaptor ligation. The processed fragments were heat denatured to single strand after purification. The single strands were circularized, and single strand circle DNA was obtained as the final library. The final library was sequenced by Illumina HiSeq 4000 platform at BGI (Shenzhen, China) to generate raw deta. The generated raw sequencing data was filtered using the program SOAPnuke [73] with default parameters to remove adapters, low-quality reads with quality value ≤ 20, to finally obtain clean reads. The quantity and quality of clean reads sequenced for each *Sorbus s.s.* sample was analyzed with FastQC v.0.11.9 [74], and the details were provided in Table 1.

### Genome assembly and annotation

The high-quality reads were used for de novo assembly to reconstruct *Sorbus s.s.* chloroplast genomes using GetOrganelle v.1.7.5.3 [75] with the reference cp genome sequence of *Torminalis glaberrima* (NC033975) with wordsize of 103 and K-mer sizes of 105 or 127 (the K-mer sizes were 105 in *S. helenae and S. tianschanica*, and were 127 in other 14 samples) and the coverage depth of the assembled genomes are provided in Table 1. Bandage software [76] was used to map all reads to the assembled cp genome sequence for visualization processing and obtaining accurate cp genomes. Complete cp genomes were annotated using the PGA program [35] with *S. insignis* as a reference, then, manually verified and corrected by comparison with five sequences in the same tribe Maleae, *Aria edulis* M. Roem. (NC045418), *Malus hupehensis*, *Micromeles thibetica* (MK920287), *Pyrus pashia* (NC034909) and *Torminalis glaberrima* using Geneious v.9.0.2 [36]. The cp genome maps were created using Organellar Genome DRAW (https://chlorobox.mpimp-golm.mpg.de/OGDraw.html). The complete cp genome sequences and gene annotation of the 16 newly assembled *Sorbus s.s.* samples were submitted to NCBI database (https://www.ncbi.nlm.nih.gov) under the accession numbers listed in Table 1. Meanwhile, all the 22 cp genomes in *Sorbus s.l.* (13 in *Sorbus s.s.*) reported previously were re-annotated.

### Genome structure and codon usage analyses

The structure, size, gene content and GC content of cp genomes were identified using Geneious v.9.0.2. LSC, SSC, IRa and IRb region were plotted with boundary positions being compared using IRscope online software (https://irscope.shinyapps.io/irapp/) [77]. All CDSs were extracted using Geneious v.9.0.2. The amount of codon and RSCU ratio was calculated using CodonW v.1.4.2 software (http://codonw.sourceforge.net/) with default parameters.

### Repeats analyses

SSRs were identified using the MISA online software (https://webblast.ipk-gatersleben.de/misa/) with the minimum repeat parameters set as 12, 6, 4, 3, 3, 3 repeat units for mono-, di-, tri- tetra-, penta-, and hexanucleotide SSRs, respectively. Online REPuter software (https://bibiserv.cebitec.uni-bielefeld.de/reputer) was used to identify and locate forward, palindromic, reverse and complement sequences with minimum repeat size of 20 bp, maximum repeat sequences number of 200 and the E-value below 1e-5.

### Comparative analyses of chloroplast genomes

To identify variable regions and intra-generic variations within *Sorbus s.s.*, the alignment was visualized using online mVISTA (https://genome.lbl.gov/vista/index.shtml) in Shuffle-LAGAN mode, with the annotated cp genome of *S. insignis* as a reference. The 16 *Sorbus s.s.* cp genomes sequences were aligned in MAFFT [78]. The alignment was used to calculate the Pi value within *Sorbus s.s.* cp genomes. The sliding window analysis was performed in DnaSP v.5 [79] with step size of 200 bp and window length of 800 bp.

### Phylogenetic analysis

The complete cp genome sequences of the 16 newly sequenced *Sorbus s.s.* with the other 48 cp genomes of tribe Maleae, one cp genomes of Amygdaleae and the outgroup (*Barbeya oleoides*) (Table S7) were aligned with the program MAFFT and any alignment issues were manually modified in Geneious v.9.0.2. Phylogenetic analyses were performed using both maximum likelihood (ML) and Bayesian inference (BI) methods based on the 63 complete cp genomes. ML analyses were implemented in RAxML v.8.0.0 [80] with GTR + GAMMA model. The best likelihood tree was obtained from 100 starting trees using rapid bootstrap analyses with 1000 bootstrap replicates. Multiparametric bootstrapping analyses with 1000 replicates was conducted to obtain the bootstrap for each node. BI analyses were conducted using MrBayes v.3.2.2 [81]. The best-fit nucleotide substitution model for BI analysis were inferred from Modeltest v.3.7 [82] and PAUP v.4.0 [83]. The Markov chain Monte Carlo (MCMC) analysis was run for 6,000,000 generations, and the trees were sampled every 1,000 generations with the initial 25% discarded as a burn-in fraction. Meanwhile, six hypervariable regions (*trnR*-*atpA*, *petN*-*psbM*, *rpl32*-*trnL*, *trnT*-*trnL*, *trnH*-*psbA* and *ndhC*-*trnV*) and the combination were uesd to reconstruct ML tree with 1000 bootstrap replicates, and compared with the tree based on complete cp genomes for the DNA barcode analysis. The resulting trees by ML and BI methods were rooted with *Barbeya oleoides* and visualized with FigTree v.1.4.3 [84].

## Supplementary Information

**Additional file 1: TableS1.** General information of 16 newly sequenced *Sorbus s.s.* chloroplast genomes

**Additional file 2: TableS2.** Genes annotated in 16 newly sequenced *Sorbus s.s.* chloroplast genomes

**Additional file 3: Table S3.** Codon usage and relative synonymous codon usage (RSCU) values of protain-coding genes of the 16 *Sorbus s.s.* chloroplast genomes

**Additional file 4: Table S4.** The comparison of SSRs among 16 *Sorbus s.s.* chloroplast genomes

**Additional file 5: Table S5.** Comparison of dispersed repeats among 16 *Sorbus s.s.* chloroplast genomes

**Additional file 6: Table S6.** The nucleotide variability (Pi) of 16 *Sorbus s.s.* chloroplast genomes

**Additional file 7: Table S7.** Information of species included in phylogenetic analyses 

**Additional file 8: Table S8.** 22 taxa in *Sorbus s.l.* with genes re-annotated 

**Additional file 9: Fig. S1** Phylogenetic tree base on *trnR*-*atpA* region resulting from the maximum likelihood (ML) analysis with Bootstrap value at nodes. **Fig. S2** Phylogenetic tree base on *petN*-*psbM* region resulting from ML analysis with Bootstrap value at nodes. **Fig. S3** Phylogenetic tree base on *rpl32*-*trnL *region resulting from ML analysis with Bootstrap value at nodes. **Fig. S4** Phylogenetic tree base on *trnT*-*trnL* region resulting from ML analysis with Bootstrap value at nodes. **Fig. S5** Phylogenetic tree base on *trnH*-*psbA* region resulting from ML analysis with Bootstrap value at nodes. **Fig. S6** Phylogenetic tree base on *ndhC*-*trnV* region resulting from ML analysis with Bootstrap value at nodes. **Fig. S7** phylogenetic tree base on 6 regions (*ndhC*-*trnV* + *petN*-*psbM*+ *rpl32*-*trnL* + *trnH*-*psbA* +* trnT*-*atpA*+ *trnT*-*trnL*) resulting from ML analysis with Bootstrap value at nodes.

## Data Availability

All 16 newly sequenced sequences in this study are available from the National Center for Biotechnology Information (NCBI) (https://www.ncbi.nlm.nih.gov; accession numbers are ON049650–ON049657, ON049659–ON049662 and ON049664–ON049667; see Table 1). Information for other cp genomes used for phylogenetic analysis download from NCBI (https://www.ncbi.nlm.nih.gov) can be found in Additional Table 7: Table S7. Voucher specimens are deposited in the Herbarium of Nanjing Forestry University (NF) and collection information was listed in Table 1.

## Abbreviations

AAs: Amino acids
BI: Bayesian inference
CNS: Non-coding sequence
Cp: Chloroplast
IRs: Inverted repeats
LSC: Large single-copy region
MCMC: Markov chain Monte Carlo
ML: Maximum likelihood
nrDNA: Nuclear ribosome DNA
Pi: Nucleotide diversity
RSCU: Relative synonymous codon usage values
SSC: Small single-copy region
SSRs: Simple sequence repeats
