# Supplementary material for: Complete chloroplast genomes of Sorbus sensu stricto (Rosaceae): comparative analyses and phylogenetic relationships
Source: BMC Plant Biol. 2022 Oct 22;22:495. doi: 10.1186/s12870-022-03858-5 (PMC9587547; doi:10.1186/s12870-022-03858-5)
Supplement: Supplementary file 9 — Additional file 9: Fig. S1 Phylogenetic tree base on trnR-atpA region resulting from the maximum likelihood (ML) analysis with Bootstrap value at nodes. Fig. S2 Phylogenetic tree base on petN-psbM region resulting from ML analysis with Bootstrap value at nodes. Fig. S3 Phylogenetic tree base on rpl32-trnL region resulting from ML analysis with Bootstrap value at nodes. Fig. S4 Phylogenetic tree base on trnT-trnL region resulting from ML analysis with Bootstrap value at nodes. Fig. S5 Phylogenetic tree base on trnH-psbA region resulting from ML analysis with Bootstrap value at nodes. Fig. S6 Phylogenetic tree base on ndhC-trnV region resulting from ML analysis with Bootstrap value at nodes. Fig. S7 phylogenetic tree base on 6 regions (ndhC-trnV + petN-psbM+ rpl32-trnL + trnH-psbA + trnT-atpA+ trnT-trnL) resulting from ML analysis with Bootstrap value at nodes. [file 12870_2022_3858_MOESM9_ESM.docx]

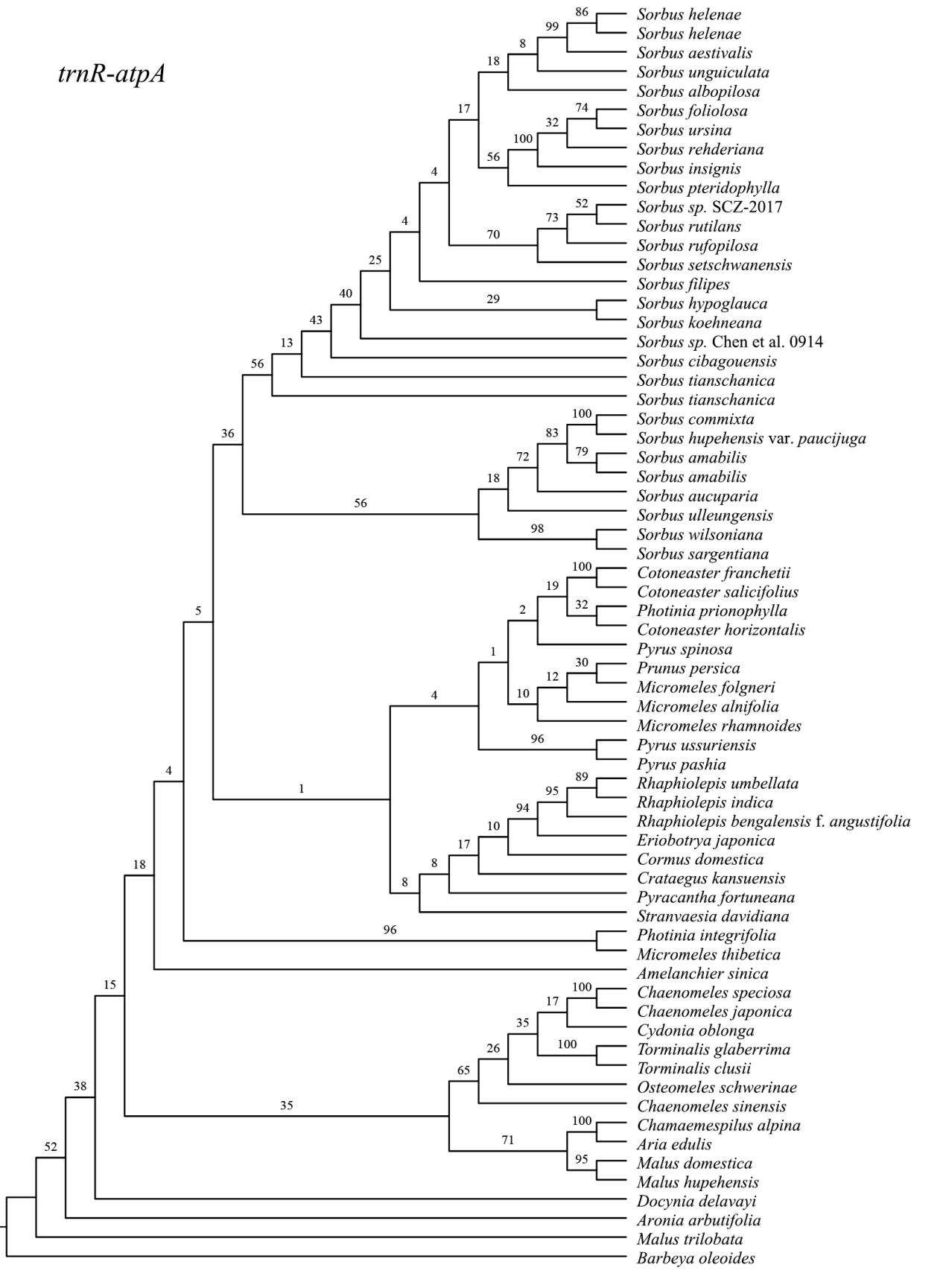


Fig. S1 Phylogenetic tree base on *trnR*-*atpA* region resulting from the maximum likelihood (ML) analysis with Bootstrap value at nodes


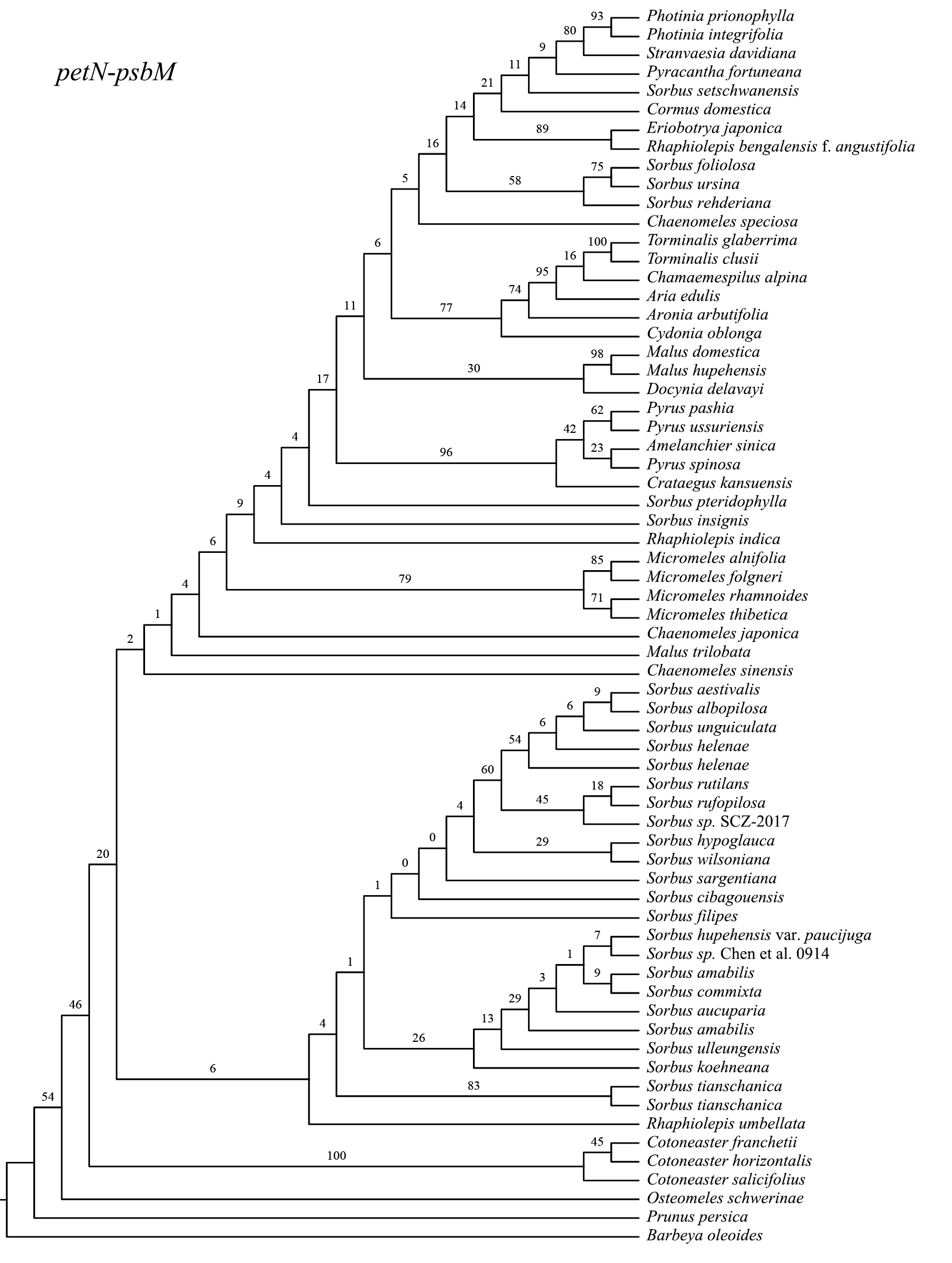


Fig. S2 Phylogenetic tree base on *petN*-*psbM* region resulting from ML analysis with Bootstrap value at nodes


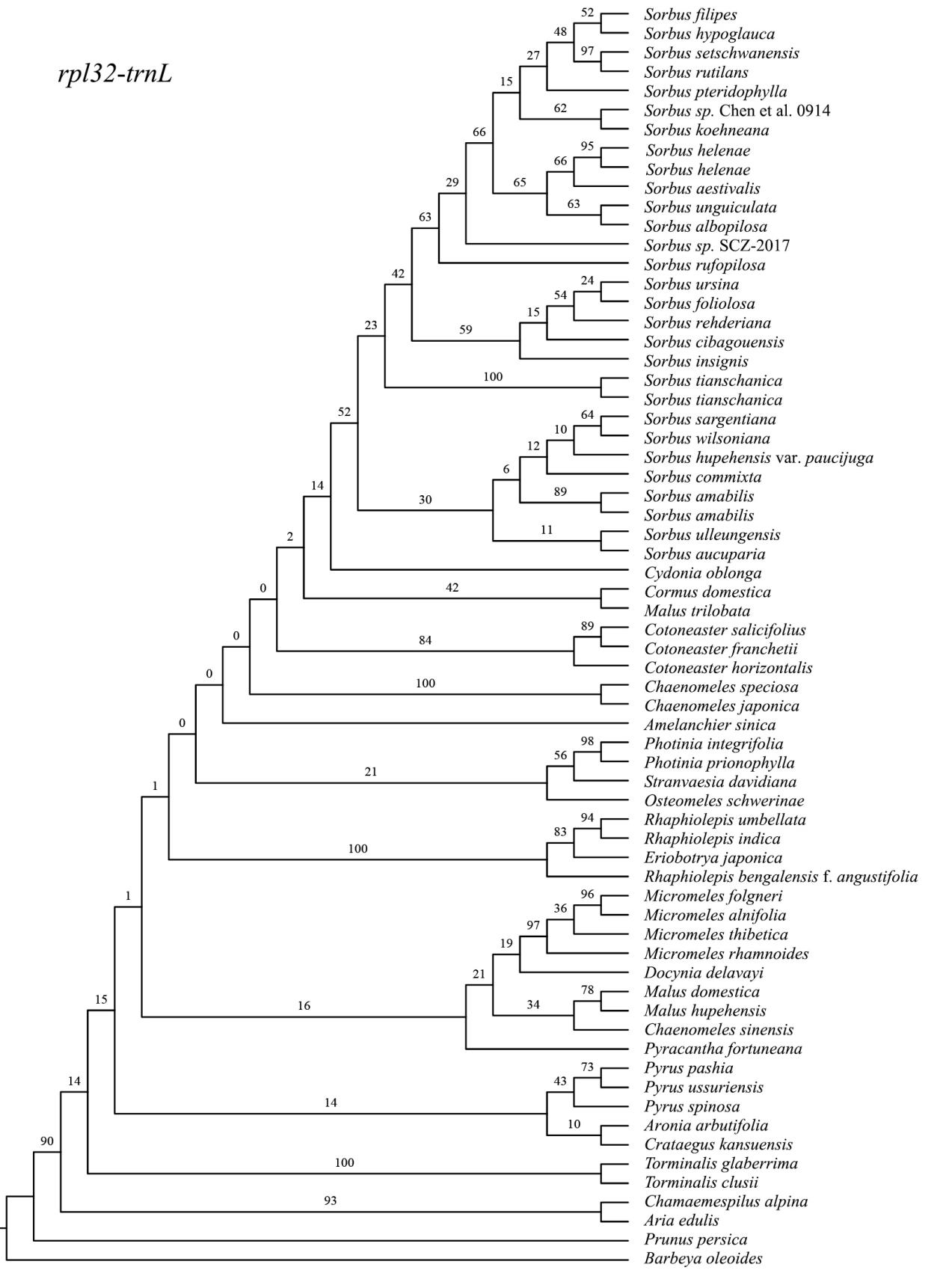


Fig. S3 Phylogenetic tree base on *rpl32*-*trnL* region resulting from ML analysis with Bootstrap value at nodes


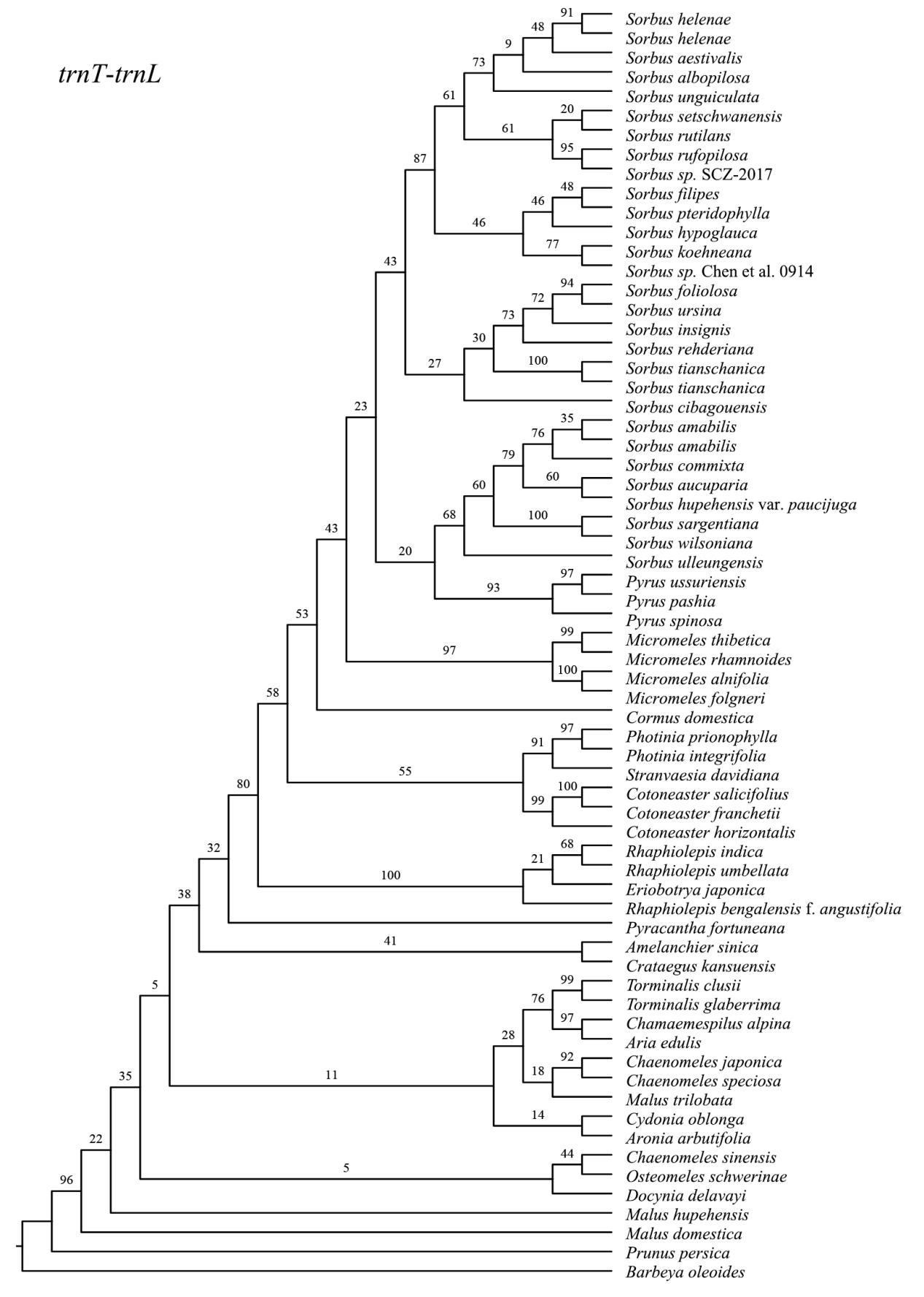


Fig. S4 Phylogenetic tree base on *trnT*-*trnL* region resulting from ML analysis with Bootstrap value at nodes


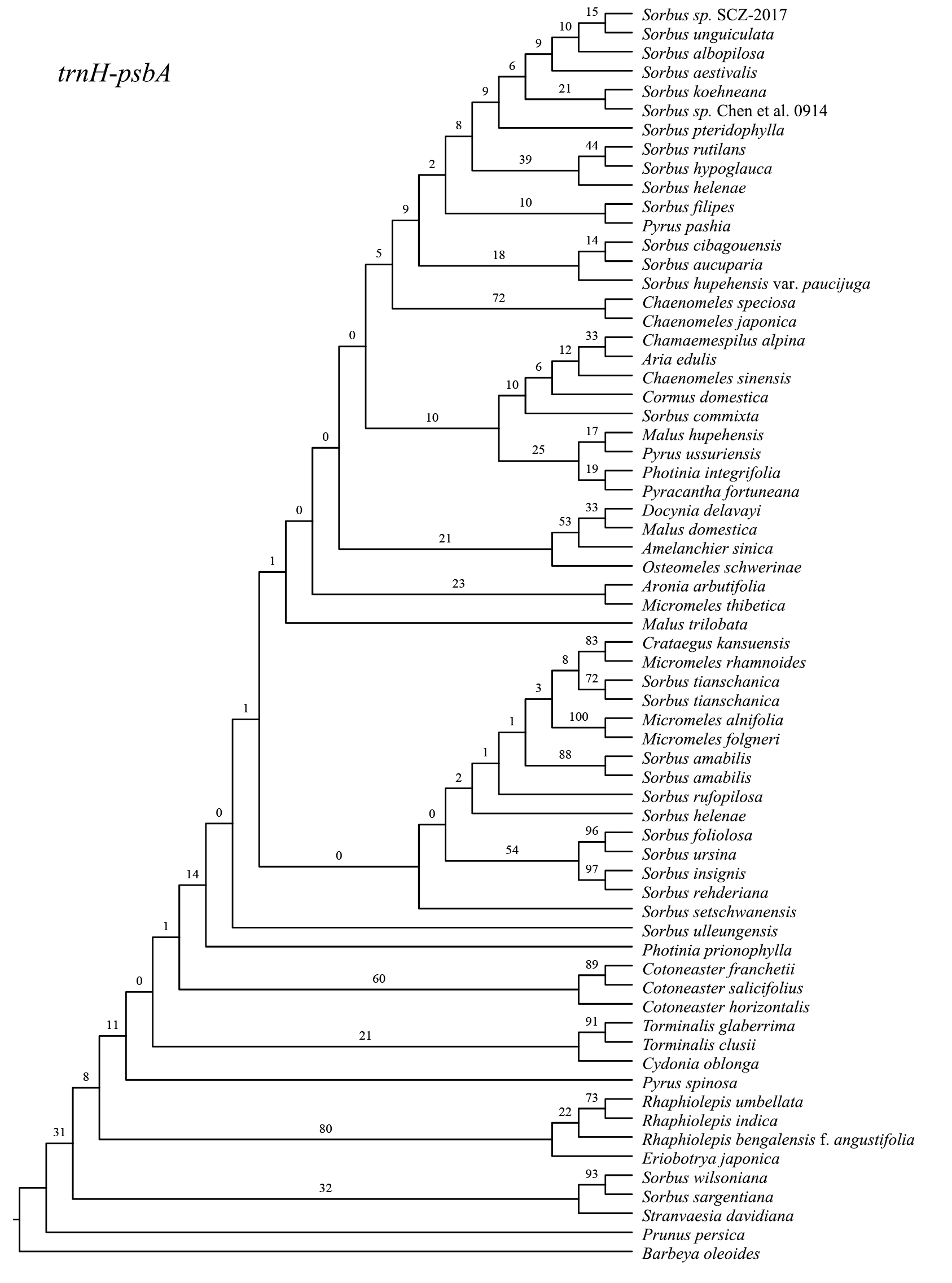


Fig. S5 Phylogenetic tree base on *trnH*-*psbA* region resulting from ML analysis with Bootstrap value at nodes


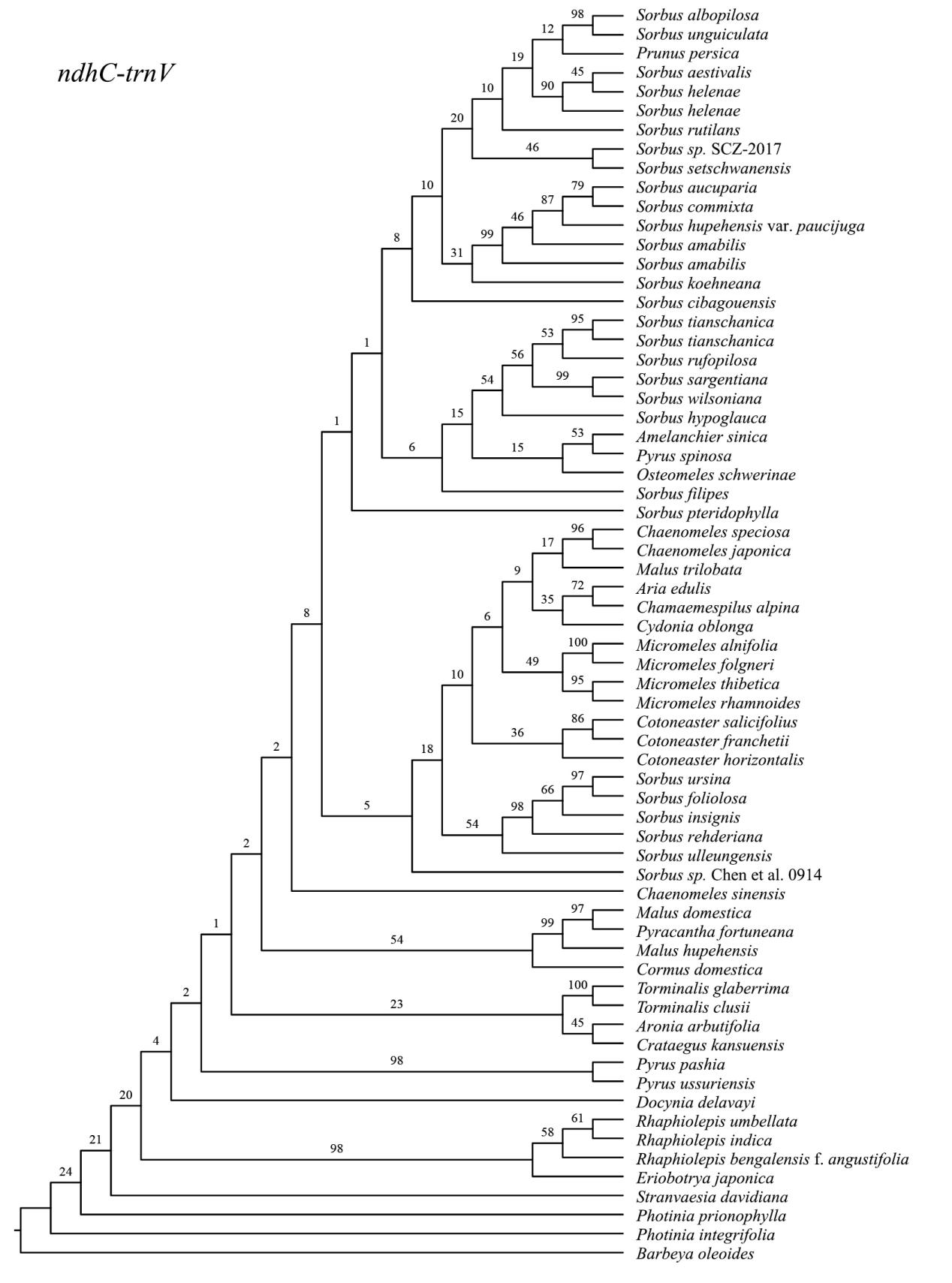


Fig. S6 Phylogenetic tree base on *ndhC*-*trnV* region resulting from ML analysis with Bootstrap value at nodes


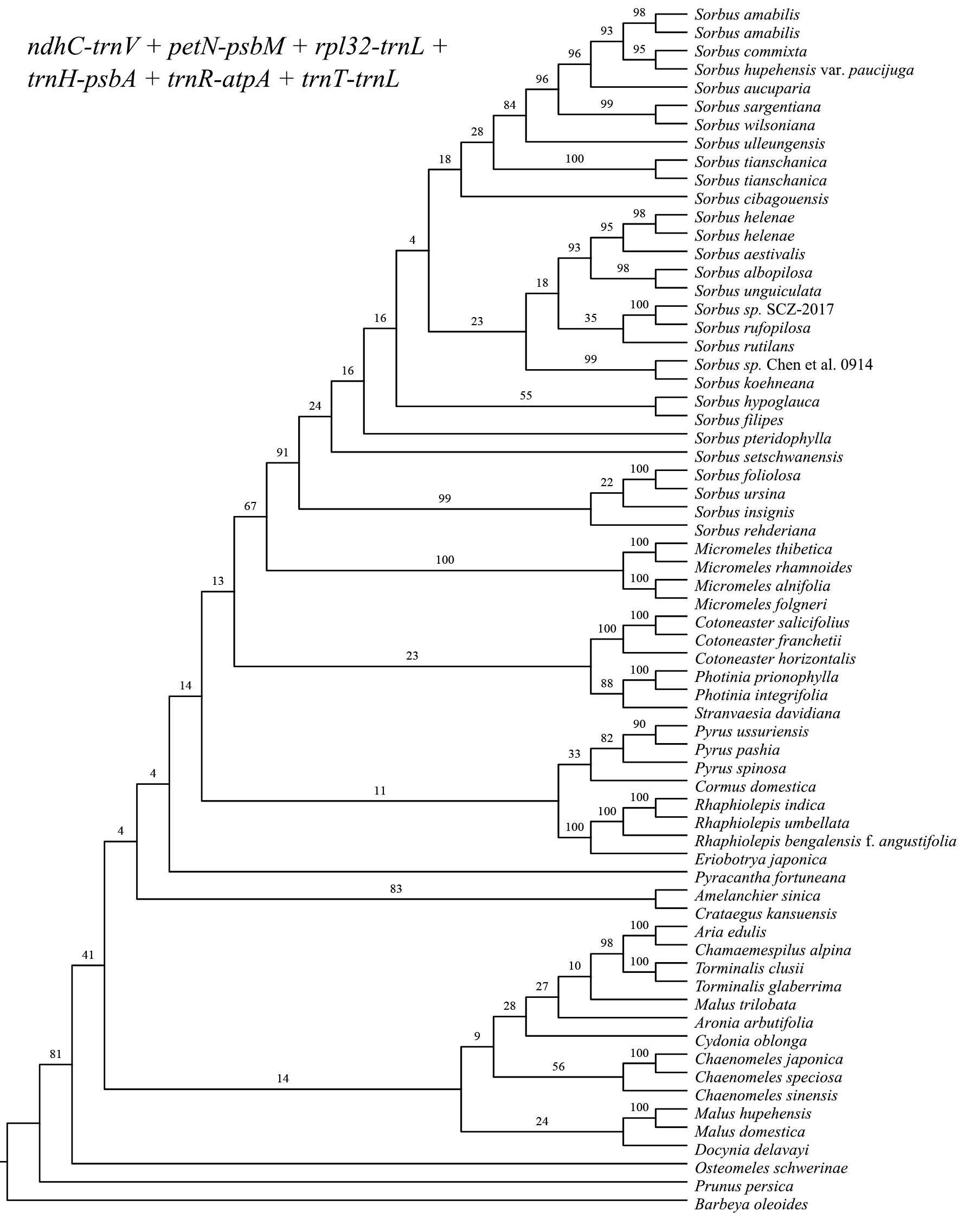


Fig. S7 phylogenetic tree base on 6 regions (*ndhC*-*trnV* + *petN*-*psbM* + *rpl32*-*trnL* + *trnH*-*psbA* + *trnT*-*atpA* + *trnT*-*trnL*) resulting from ML analysis with Bootstrap value at nodes
